# Sensitization pattern to inhalant and food allergens in symptomatic children at first evaluation

**DOI:** 10.1186/s13052-015-0204-9

**Published:** 2015-12-08

**Authors:** Alessandro Fiocchi, Valentina Pecora, Carl Johan Petersson, Lamia Dahdah, Magnus P. Borres, Maria J. Amengual, Johannes Huss-Marp, Oscar Mazzina, Francesco Di Girolamo

**Affiliations:** Pediatric Hospital Bambino Gesù, Vatican City, Italy; Thermo Fisher Scientific, Uppsala, Sweden; Department of Women’s and Children’s Health, Uppsala University, Uppsala, Sweden; Corporació Sanitària Parc Taulí, Sabadell, Spain; Allergy Research Group, Department of Dermatology, University of Freiburg, Freiburg, Germany

**Keywords:** Allergy, Children, IgE, Prevalence, Primary Care, Sensitization

## Abstract

**Background:**

Data on specific IgE sensitization prevalence in children with allergy-like symptoms seen in the primary care setting are rare. Early diagnosis of allergic diseases is important to prevent clinical manifestations, exacerbations or expansion of allergic diseases to other organ systems. The present study aims to assess the usefulness of early serological diagnosis in children with common allergic symptoms.

**Methods:**

532 children (<15 years of age), with at least one of ten allergy-like symptoms, from 21 primary care centers in two geographic areas of Italy and Spain were included in the study. Patients were tested with, either Phadiatop® Infant (0–5 years of age) or Phadiatop® and food mix (fx5e) (>5 years of age) to discriminate atopic from non-atopic subjects. A blood sample of atopic subjects was taken for additional 6–26 specific IgE antibody determinations from a predefined panel using the ImmunoCAP^®^ System.

**Results:**

267 children (50.2 %) were positive in the initial test and were classified as atopic. 14 % were mono-sensitized, 37 % were sensitized to 2–3 allergens and 49 % to more than 3 allergens. The average number of symptoms in the atopic group was 3.3 vs 2.8 in the non-atopic group. The prevalence of sensitization to single allergens was highest for grass and ragweed pollen and house-dust mites (19–28 %). Sensitization to tree allergens was highest for olive tree (16.5 %). Cow’s milk and egg white were the most sensitizing foods (~15 %). Food allergen sensitization predominated in younger children (OR = 2.8) whereas the inverse occurred with inhalant allergens (OR = 2.5 to 5.6). A significant positive correlation between patient age and the number of sensitizations was found.

**Conclusions:**

Specific IgE sensitization in children with allergy-like symptoms is common. Multiple sensitization is predominating. Number of clinical symptoms was higher in the atopic group compared to the non-atopic without a correlation with the number of positive allergens. Age seems to play a crucial role in the development of sensitization with a significant positive correlation between patient age and the number of sensitizations.

## Background

In the primary care setting clinicians are frequently faced with allergy-like symptoms which need to be etiologically assessed to ensure correct diagnosis and patient management. Since allergic sensitization is the prerequisite for allergic disease, it is the key diagnostic assessment in this context [1]. However, sensitization in children do not univocally correspond to symptomatic allergic disease [2].

In the pediatric population seen in the primary care setting allergy-like symptoms are frequent [3, 4]. Cough, blocked or runny itchy nose, sneezing and impaired sleep, the most common causes of outpatient consultations, are typical signs and symptoms of allergic as well of infectious respiratory disease [5]. Children with early development of IgE sensitization to cow’s milk and hen’s egg proteins or early sensitization to inhalant allergens carry an increased risk for later development of respiratory allergic diseases [6]. Those with earlier onset of sensitization to grass pollen allergens carry an increased risk to develop allergic rhinitis compared to children with a later onset of sensitization [7]. For these reasons, the identification of allergic conditions is important, since early diagnosis of allergic disease may provide opportunities to prevent the later development of asthma.

Data on allergic sensitization in the general pediatric population are widely available. Large cross-sectional studies report sensitization prevalence rates of 40 % in the German 3–17-years old [8], 44 % in American above the age of six [9], and 40 % in the Netherlands [10]. These rates increase with age: sensitization to airborne allergen has been reported in a Swedish cohort to raise from 17 % at the 4 years to 45 % at 16 years [11].

These figures are far less studied in children presenting with allergy-like symptoms in the pediatric primary care who have not received an allergy diagnosis so far.

The aim of this study is to provide insight into sensitization rates, levels and patterns in children with common allergic symptoms without an allergy diagnosis to support the clinician in his decision-making process.

## Methods

### Patients and study design

Data collected in a previously published study was used for this survey [12]. Out of 721 patients included in the original study, 532 children from 21 primary care centres in two geographic areas of Italy (Lombardy) and Spain (Catalonia) were included in the present study. Children having a minimum of one of the following ten allergy-like symptoms present at any time during the last year were candidates for inclusion, (1) Wheezing in the chest; (2) Shortness of breath not correlated to physical exercise; (3) Long-standing cough; (4) Itchy throat; (5) Recurrent symptoms of a cold; (6) Recurrent sneezing; (7) Runny or blocked nose when not having a cold; (8) Eczema; (9) Urticaria and (10) Angioedema. A previously known allergy diagnosis and/or previous specific IgE/SPT results were criteria for exclusion as well as known HIV or hepatitis infection. A blood sample was taken from each included child for specific IgE antibody determination.

Study information was given to each participant and an informed consent was signed by a legal guardian. The protocol was reviewed and approved by the ethics comitee (Comité Étic d’Investigació Clinica de la Fundació Jordi Gol i Gurina) in Barcelona on February 28th 2002 prior to the initiation of the study.

### IgE analysis

Serum samples were analysed for IgE antibodies using the ImmunoCAP^®^ system according to the manufacturer’s guidelines (Thermo Fisher Scientific (formerly Pharmacia Diagnostics), Uppsala, Sweden). First, all children were tested with either Phadiatop^®^ Infant (0–5 years of age) or Phadiatop® and food mix (fx5e) (>5 years of age). These are assays for the graded determination of atopy with semiquantitative and qualitative results. If a positive result was found, additional 6–26 specific IgE tests from a predefined panel were performed. Specific IgE levels >0.35 kU_A_/l were regarded as a positive test result. Depending on the geographical origin (Italy or Spain), the panel of the additional atopy-related allergens has provided the determination of different foods, pollens, moulds, dust mites and pet dander. Details of all allergens tested are shown in Tables 1 and 2.

**Table 1:** Test algorithm, allergens, number of patients and prevalence figures

Initial IgE test (cut off= > 0.35 kU_A_/L)	Number of patients/^a^ of positive patients *(%)*		
	Italy	Spain	Total
<5 years of age: Phadiatop® Infant ImmunoCAP^TM^	172/81 *(47)*	76/14 *(18)*	248/95 *(38)*
≥5-15 years of age Phadiatop® ImmunoCAP^TM^ + fx5E	172/109 *(63)*	112/63 *(56)*	284/172 *(61)*
All patients	344/190 *(55)*	188/77 *(41)*	532/267 *(50.2)*
Patients by sex (Italy and Spain)	Girls (% pos)	Boys	
	232 (43.6)	300 (56.4 %)	

^a^positive/(#actual tested + #negatives at initial test). Per country or per both countries together (weighted) depending on if test was performed in only one or in both countries

**Table 2:** Test algorithm, allergens, number of patients and prevalence figures

Following-up allergens (if initial IgE test was positive)						
	Country prevalence % (^b^tested)		Prevalence and 95 % CI %^a^ and total^b^ tested (a) (>0.35 kU_A_/L)			Group/ Cluster (see Fig. 3)
	Italy	Spain				
						Grass
Velvet grass (*g13*)		n.d.	28.4	(23.6–33.6)	(170)	X
Rye grass (*g5*)	28.1 (170)	13.2 (70)	22.8	(19.2–26.7)	(240)	X
Timothy (*g6*)	27.7 (170)	12.6 (71)	22.3	(18.8–26.2)	(241)	X
Bermuda grass (*g2*)		n.d.	21.5	(17.2–26.4)	(171)	X
						Weed
Common ragweed (*w1*) *(Ambrosia*		n.d.	21.2	(17.0–25.9)	(190)	X
*artemisiif.)* Wormwood (*w5*) *(Artemisia absinthium)*		n.d.	11.7	(8.4–15.7)	(170)	X
Wall pellitory (*w19*) *(Par. Officin.)*		n.d.	10.2	(7.1–14.0)	(170)	X
Wall pellitory (*w21*) *(Par. judaica)*	n.d.		7.7	(4.9–12.6)	(70)	-
Mugwort (*w6*) *(Artemisia vulgaris)*	n.d.		7.2	(3.9–12.0)	(70)	-
Goosefoot (*w10*) *(Chenopodium album)*	n.d.		3.9	(1.6–7.8)	(71)	-
						Hdm
Mite (*d1*) *(dermatophagides pteronyssinus)*	19.1 (171)	19.7 (71)	19.3	(16.0–23.0)	(242)	X
Mite (*d2*) *(dermatophagides farinae)*	20.1 (170)	16.4 (71)	18.8	(15.5–22.5)	(241)	X
						Tree
Olive (*t9*)	16.4 (169)	16.5 (70)	16.5	(13.3–20.0)	(239)	X
Hazel (*t4*)		n.d.	12.7	(9.2–16.8)	(170)	X
Birch (*t3*)	13.9 (170)	3.8 (70)	10.3	(7.8–13.2)	(240)	X
						Food
Milk (*f2*)	19.4 (129)	5.5 (33)	14.7	(11.5–18.5)	(162)	X
Egg (*f1*)	17.3 (129)	9.0 (33)	14.5	(11.3–18.2)	(162)	X
Wheat (*f4*)	12.0 (129)	5.5 (33)	9.8	(7.2–13.1)	(162)	X
Fish (*f3*);	0.0 (129)	0.0 (33)	0		(162)	X
Peanut (*f13*)	9.2 (128)	6.3 (33)	6.6	(4.6–9.0)	(161)	X
Soya bean (f14)	7.1 (128)	4,9 (33)	5.1	(3.3–7.3)	(161)	X
Carrot (*f31*)		n.d.	7.0	(3.7–11.9)	(82^b^)	-
Tomato (*f25*)		n.d.	5.8	(2.8–10.4)	(82)	-
Potato (*f35*)		n.d.	5.2	(2.4–9.7)	(82)	-
Rice (*f9*)		n.d.	4.1	(1.7–8.2)	(82)	-
Meat (*f27*)		n.d.	1.7	(0.4–5.1)	(82)	-
						Pets
Dog (*e5*)	n.d.	10.4 (72)	10.4	(6.4–15.8)	(72)	X
Cat (*e1*);	7.7 (169)	9.3 (72)	8.3	(6.0–11.1)	(241)	X
Cockroach (*i6*)	n.d.		1.1	(0.1–3.9)	(70)	-
						Moulds
*Alternaria alternata* (*m6*)	6.8 (170)	7.7 (70)	7.1	(5.0–9.7)	(240)	X
*Aspergillus fumigatus* (*m3*)	3.5 (344)	n.d.	3.5	(1.8–6.0)	(190)	X
*Cladosporium herbarun* (*m2*)	1.9 (170)	2.2 (70)	2.0	(0.9–3.6)	(240)	X

^a^Based on number of patients with allergy-ike symptoms included in Spain and/or Italy centers

^b^Performed in patients <5 years of age

### Statistics

All statistical analyses were performed using the SAS^®^ statistical software system version 9.3 (SAS Institute Inc., Cary, US). All statistical tests were two-sided and a significance level of <0.05 was regarded as statistically significant. The chi-square test or, when appropriate, Fischer’s exact test were used to compare proportions. Mann–Whitney-nonparametric test was used to compare the atopic and non-atopic group on the basis of clinical symptoms referred. Odds ratios with 95 % confidence interval were calculated using SAS: Proc Freq / chisq relrisk option.

## Results

532 subjects were enrolled in the present study, of which 248 were children less than 5 years and 284 between 5 and 15 years of age with a median age of 5.3 years (2.8 in children <5 years of age and 8.4 in older children). Considering the entire study population a higher percentage of males (56.4 %) than females (43.6 %) was noticed (Tables 1 and 2). 267 children were positive in the initial test and were classified as atopic. The prevalence of males (58.8 % [95 % CI 46.5–62.1]) was confirmed in atopic population characterized by an overall prevalence of 50.2 % (45.9–54.5).

In the second diagnostic work-up, 95 (35.6 %) children were younger than 5 years of age and 172 (64.4 %) were between 5 and 15 years of age. They were all tested for in average 14.6 allergens [SD 6.5; range 1–28]. In this second part 18 children were negative in all of the specific IgE tests but initially they were classified as atopic. Finally, there were 249 children (46.8 %) that were sensitized to at least one of the allergens included in the additional panel [mean 5.3; SD 4.6; range 1–24].

**Fig. 1:**
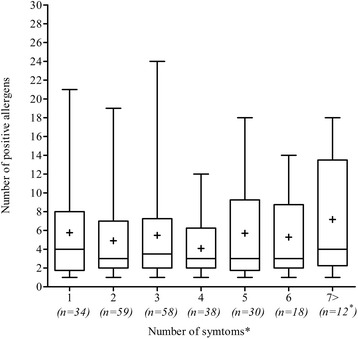
*Number of allergen sensitizations in regard to number allergy-ike symptoms recorded.* Boxes include median (line) and mean (+) values and the interquartile range (25-75 %). Whiskers extend to most extreme data points. Spearman Correlation Coefficient, r = 0.01517; *p =* 0.8117 (ns). n is the number of patients, * includes 2 patients with 8 symptoms

The average number of allergy-ike symptoms was significantly higher in the atopic group compared to the non-atopic group, 3.3 vs 2.8 symptoms (*p =* 0.005). In the atopic group the most frequent respiratory symptom was runny/blocked nose when not having a cold (54 %, 95 % CI 48–60) followed by recurrent symptoms of a cold (49 %, 43–56) while eczema was the most frequent skin symptom (36 %, 30–42). No correlation between number of clinical symptoms and the number of positive allergens was found (Fig. 1).

Amongst the atopic children, 14 % (95 % CI 10–19) were monosensitized, 37 % (30–43) were sensitized to 2 up to a maximum of 3 allergens and 49 % (43–56) to more than 3 allergens. The prevalence of sensitization to single allergens was highest for velvet grass and ragweed pollen and house-dust mites (19–28 %). Among the moulds tested, Alternaria alternata was the most prevalent allergen (7 %). Sensitization to tree allergens was highest for olive tree (16 %). For birch pollen the sensitization was much higher in the Italian part compared to the Spanish part 13.9 % VS 3.8 %) (Tables 1 and 2). Among pets, dog was the most common sensitizer (10 %). Cow’s milk and egg white were the most frequent sensitizing foods ~15 % (19.4 % It and 5.5 Sp) and ~15 % (17.3 % It and 9.0 % Sp) respectively followed by wheat ~10 % (12.2 % It and 5.5 Sp). None of the 162 tested children had antibodies to fish. Regarding the sensitization to animals, dog was the most common allergen (10 %).

**Fig. 2:**
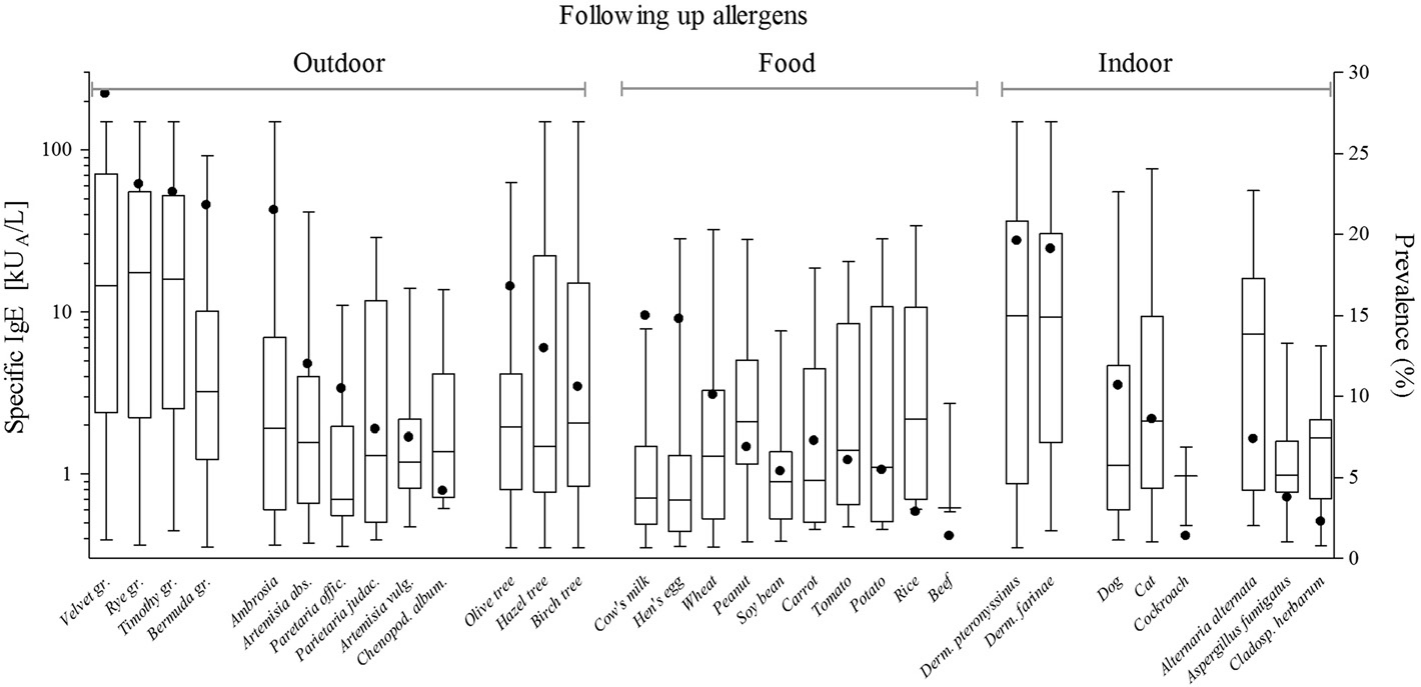
*sIgE sensitization levels (kUA/L) of positive samples in correlation to the prevalence of sensitization.* Left Y-axis: Boxes include median (line) and the interquartile range (25-75 % observation). Whiskers extend to the most extreme data points. Right Y-axis: Black dots shows the prevalence for the allergens

A correlation between the mean specific IgE concentration and sensitization prevalence was noticed only for grass pollen and house dust mites (Fig. 2). Regarding other allergens tested, no similar correlation was found. Sensitization to ragweed was prevalent (21 %) but the mean concentration was only 2 kU_A_/L. On the other hand, Alternaria sensitized patients showed a prevalence of 7 % despite a mean sIgE concentration of 9 kU_A_/L. The food sensitization was in general characterized by low levels of specific IgE, where the peanut sensitized children had a mean concentration of 2 kU_A_/L.

**Fig. 3:**
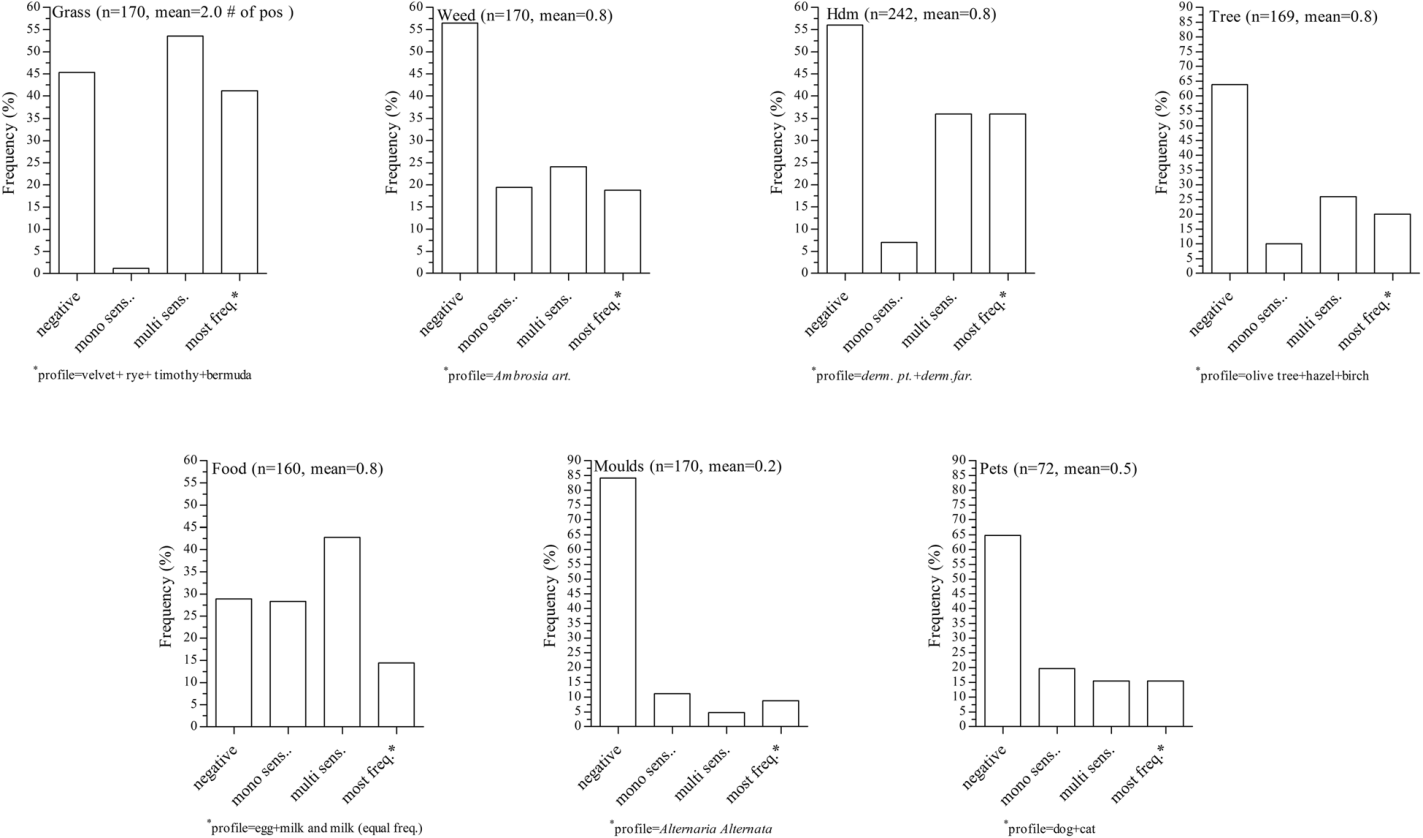
*Patients profile in seven groups of selected allergens.* Proportions of negative results, mono sensitization and multiple sensitizations as well as the most frequent sensitization profile are reported. Within brackets number of observations and mean number of positives results are given. See Tables 1 and 2 under Group/cluster for allergens included, indicated with X

In Fig. 3 the patients profile in seven groups of selected allergens (grass, weed, house dust mite, tree, food, moulds and pets) was analysed by focusing on mono- or multiple sensitization and most frequent sensitization profile within the each single group. Monosensitization was most prevalent within the food allergen group. Thirty percent were mono-sensitized to one of the six food allergens as reported in Tables 1 and 2. Within the grass group, monosensitization was extremely rare (2 %), while multisensitization was prevalent (53 %) and characterized by the combination of velvet-, rye-, timothy- and Bermuda grass.

Cosensitization to moulds was common in children sensitized to pollen with likelihood odds ratios with 95 % confidence interval ranging from 2.33 to 3.44 (Table 3).

**Table 3:** Likelihood of being sensitized in pairwise combinations. Crude odds ratio with 95 % confidence interval

Cluster	Grass	Weed	Hdm	Tree	Food	Pet	Mould
*Grass*	-	12.3^***^ *(6.42-23.7)*	0.81^a^ *(0.48-1.34)*	10.2^***^ *(5.43-19.1)*	0.86^a^ *(0.41-1.80)*	1.51^a^ *(0.81-2.83)*	2.33^*^ *(1.16-4.71)*
*Weed*		-	1.51^a^ *(0.89-2.55)*	11.7^***^ *(6.30-21.8)*	2.54^*^ *(1.09-5.90)*	1.16^a^ *(0.62-2.19)*	2.72^**^ *(1.37-5.41)*
*Hdm*			-	0.85^a^ *(0.50-1.45)*	1.10^a^ *(0.49-2.44)*	2.70^**^ *(1.42-5.11)*	1.62^a^ *(0.82-3.18)*
*Tree*				-	2.31^a^ *(1.01-5.24)*	2.34^**^ *(1.25-4.39)*	3.44^***^ *(1.71-6.93)*
*Food*					-	1.89 ^a^ *(0.65-5.45)*	1.17 ^a^ *(0.42-3.24)*
*Pet*						-	2.61^*^ *(1.26-5.43)*
*Mould*							-

^a^not significant, * p ≤ 0.05, ** p ≤ 0.01, ***p ≤ 0.001

**Fig. 4:**
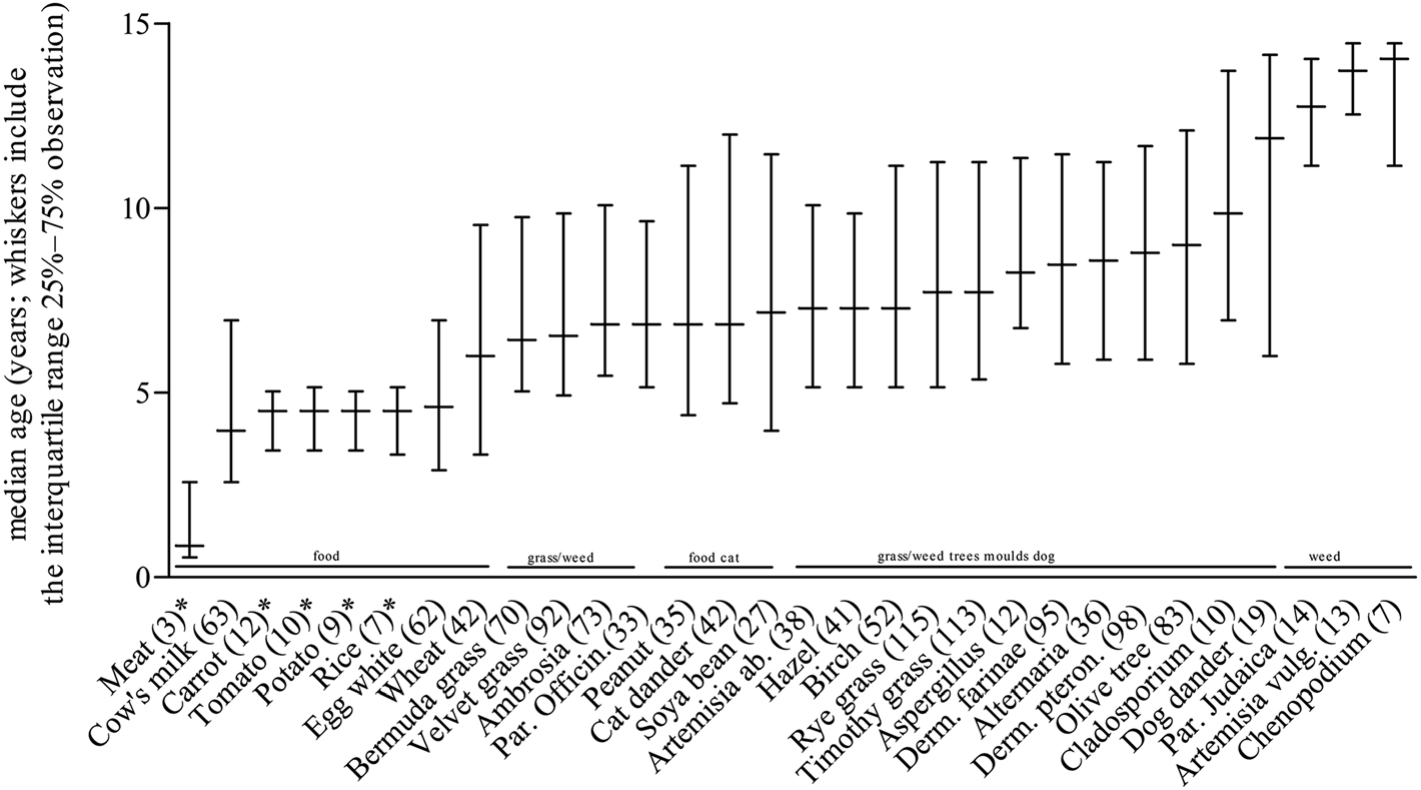
*Sensitization development and patient’s age.* Number of positive observations with the cut-off over 0.35 kUA/L correlated with the patients age; * allergens that are not included in the OR since these sIgE tests were only performed in children <5 years of age)

Comparing the age of the enrolled subjects with the sensitization to each single allergen (Fig. 4), we observed that younger children were more likely to be sensitized to food allergens than older children. A sensitization to grass, weed and tree pollen as well as to house dust mite, pets and moulds was more marked in children older than 6 years of age. The likelihood odd ratio of sensitization to food allergens was 2.3 (95 % CI 1.1–4.9) in children younger than 5 years of age. Considering the children aged 5 to 15 years and sensitized to inhalant allergens, the likelihood odd ratios were for grass 2.8 (1.6–4.8), weed 3.5 (1.9–6.3), tree 3.2 (1.6–6.3), house dust mite 5.6 (3.0–10.1), pet 2.5 (1.2–5.0) and mould 2.6 (1.2–5.7). An interesting finding is that the median age for cat sensitization was 6 years of age compared to 10 years of age for dog sensitization.

**Fig. 5:**
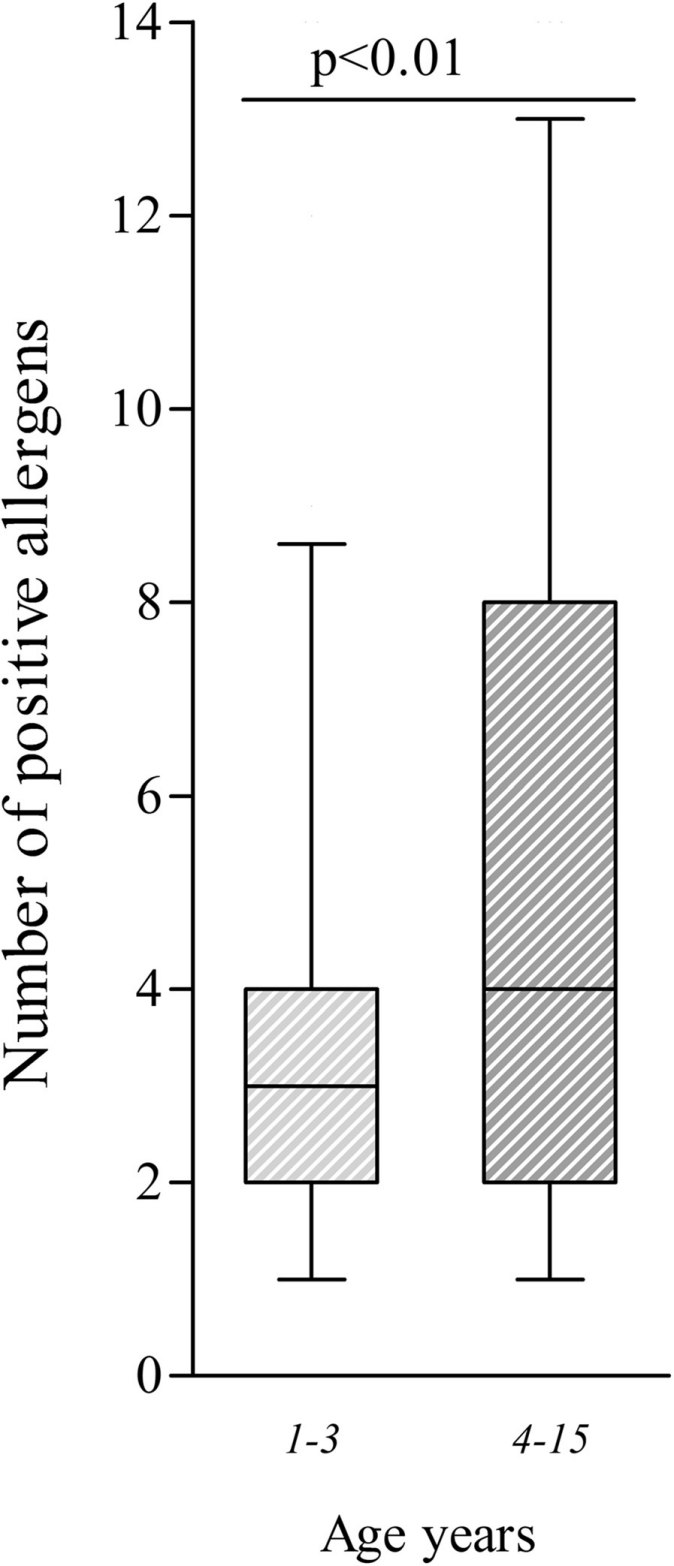
*Relationship between multiple sensitization and patient’s age.* Median and interquartile range (25-75 %) of sensitizations in 63 (1–3 years of age) and 186 (4–15) children respectively. Whiskers extend to the 10–90 percentile. *p <* 0.01 (Mann Whitney test)

In Fig. 5 numbers of sensitizations in different age groups are shown. When comparing the 1–3 years group with the 4–15 years group there was a statistical difference—median value of sensitization in the 1–3 years group was 2.5 compared to 4 in the 4–15 years group (*p <* 0.01).

## Discussion

This study shows that sensitization to multiple allergens is common among children with allergy-like symptoms in pediatric primary care. Our data analysis led to three key findings: (i) polysensitization was most common for inhalant allergens, while monosensitization for foods. (ii) No correlation was found between the number of symptoms and sensitizations which (iii) showed a significant increase with age.

Children with symptoms of allergic diseases are usually managed by primary care pediatricians and general practitioners, although the available data are reported only by a limited number of studies. Our findings on prevalence, levels and patterns of sensitization will be discussed in the light of the epidemiological data available so far.

In regard to prevalence we found that 47 % of the individuals in our study were sensitized to at least one of the 6–26 specific IgE tests from a predefined panel, while over 50 % showed a positive reaction to the first screening performed with a multiallergen serum test containing important food- and aeroallergens. In regard to the general pediatric population a range of sensitization rates have been reported, but the data appear to be influenced by sample size, recruitment pattern, geographic distribution, as well as age, gender and allergy tests performed.

Wickman et al. found in Sweden the above mentioned sensitization rates to airborne allergens between 17 % and 45 % [11]. In a study from Korea [13] a prevalence of sensitization of 47.9 % in an unbiased population of nearly 8000 schoolchildren was detected with skin prick test performed with 19 common allergens. In a recent study based on nearly 13000 German children from the general population aged 3–17 years, sIgE sensitization to at least 1 of 20 tested allergens was detected in 40 % of the participants [8]. Salo et al. found a sensitization rate of 44 % based on the investigation of 9440 children aged 6 years and older in a US population study (NHANES) by using a panel of 19 allergens. In young children, aged 1 to 5 years a lower prevalence of 36.2 %, was seen in the same population [9]. Additional prevalence studies were performed in a population of 1700 children aged 7–8 years by Rönmark et al. [14], who found a prevalence of 30 % due to the positive result from skin prick test with 10 common allergens. Similar results were obtained by Mortz et al. [15] in 1500 adolescents aged 12–16 years in Denmark showing a sIgE sensitization prevalence of 29.6 %. Investigating pediatric patients from general practitioners and hospital based specialists de Jong et al. found that 40 % had one or more positive specific IgE test of which 31 % were mono-sensitized [10].

Even though it is virtually impossible to perform a true statistical comparison of our study with the data published so far due to substantial methodological differences of several important parameters an overall pattern can be identified. The above mentioned sensitization rates range between 17 % and 47.9 % in the general pediatric population as well as in unselected pediatric patients and thus are in average lower than in our cohort. Age is positively correlated with the sensitization prevalence which tends to be higher in older children and young adults and therefore needs to be taken into account when comparing the data from a range of studies. Furthermore a different number of allergens have been tested throughout the investigations additionally explaining different findings.

Our study data shows that allergic sensitization is more prevalent in children with allergy-like symptoms compared to the general pediatric population which is unselected concerning the occurrence of signs and symptoms of allergic disorders. This finding is not surprising for the clinician and underlines the importance of the allergy workup in this group of patients seen frequently in primary care.

Consequently in children with doctor-diagnosed allergic disease higher sensitization rates are found as has been shown by Patelis et al. [16]. They have recently analysed an asthma cohort with subjects aged 10–35 and sensitization prevalence to grass of 54 % and dog 60 % which is considerable higher than in our cohort (28 % and 10 % respectively). Taking into account that almost half of our patients were below the age of 5 years and none of them older than 15 years this finding is in line with what was stated above concerning the influence of age. Additionally the doctor-diagnosed asthma fell within the inclusion criteria influencing the sensitization rates of the study. Geographical difference, recruitment process and the number of allergens tested have to be taken into account for a thorough comparison.

Besides the prevalence of sensitization it is important to acknowledge the specific IgE levels found in our cohort for the different allergens. The high prevalence of sensitization was correlated to high average sIgE-levels only for some allergens tested such as for different grasses (e.g. velvet grass, rye grass). A different trend was observed for others such as hen’s egg and cow’s milk with a high prevalence combined with low sIgE-levels. These observations can be due to a multitude of factors including age but also the underlying molecular basis of allergy. We found in our cohort that both wheat and peanut sensitization was quite common with prevalence rates of 9.8 % and 6.6 % respectively. The mean sIgE level for these two allergenic sources was quite low and the results could very well be a result of cross reactivity with birch and grass pollen. Molecular-based allergy diagnostics is an approach used to map the allergen sensitization at a molecular level, using purified natural or recombinant allergenic molecules (allergen components) instead of allergen extracts [17]. This approach useful in identifying genuine versus cross-reactive sensitization in poly-sensitized patients. Even though we did not perform molecular allergy diagnostics in this study, we were able to analyze (data not shown) our prevalence data of peanut (6.6 %) in combination with and without sensitization to grass and/or birch pollen in accordance with the study of Niggeman et al. [18]. We found that the peanut sensitization in the absence of sIgE positive to grass and birch pollen was only 0.6 % and 1.3 respectively. In coherence with the findings by Niggeman, we conclude that the 6.6 % prevalence of peanut sensitization might be explained by cross-reactivity to pollen and that the true prevalence of peanut sensitization is lower and within the range as reported by Hourihane and Sicherer [19, 20]. For this reason the patterns of sensitization found in our cohort have to be considered in regard to the molecular principles of allergy. Our data on the likelihood of being sensitized in pairwise combinations showed highly significant correlations (p ≤ 0.001) between trees and grass, weeds and moulds, as well as between grass and weeds. Even though our findings in regard to polysensitizations have to be evaluated considering the possible allergen cross-reactivity. Polysensitization was found in more than half of the patients sensitized to grasses, while monosensitization was most frequent for food allergens. However, also in patients sensitized to food allergens polysensitizations could be observed in more than 40 % of cases suggesting different underlying modes of sensitization.

In the current study a large difference in the prevalence of food sensitization between the Italian and Spanish groups was found probably due to that the children in the Italian group were much younger than the corresponding Spanish group; 6.1 vs 9.1 years of age.

Data from our study cohort showed how sensitization patterns are strongly age dependent with a clear shift from food allergens in early childhood to aeroallergens in older children. This phenomenon, well described in the literature, underlies the importance of allergy diagnostics in the primary care setting. The allergy march is not confined to a shift from one atopic disease and allergen group to another, but includes also increasing complexity of sensitization profiles which was clearly documented in our study with the significantly higher number of sensitizations in children aged 4 to 15 years compared to children between 1–3 years of age [21]. This observation is supported by the principle of molecular spreading reported by Hatzler et al. [22] who showed for timothy grass pollen sensitization that the number of *Phl p* allergens recognized by IgE antibodies increase with patient age. Along the same line Willumsen et al. [23] have described how intra-molecular epitope spreading represents the reason for the progression from low-complexity to full-complexity IgE repertoires.

At present, the identification of a child at risk to develop a clinically manifestation of allergic disease is not possible with certainty [6]. Current research points to individual indicators such of history of allergic symptoms, early and severe sensitization to food allergens (especially eggs) and aeroallergens as well as early viral infection associated with wheeze and adverse environmental exposures. This means that the primary care physician has to interpret the sensitization test results in relation to these factors in the context of the observed symptoms of allergic diseases.

In order to interpret sensitization data and assess clinical relevance, not only history of allergy-like symptoms has to be taken into consideration but also the allergic family history. Hatzler et al. [7] have recently documented that parental hay fever and specific IgE to grass/or birch pollen are strong pre-clinical determinants and potentially good predictors of seasonal allergic rhinitis. Their finding on the onset of sensitization as risk factor to develop allergic rhinitis could be interpreted as a stronger genetic predisposition which leads to an early onset on sensitization and to a higher risk to develop disease after onset of sensitization. This means that children sensitized to grass or birch pollen but not fulfilling the criteria of allergic rhinoconjuntivitis diagnosis, should be carefully monitored in terms of disease development.

The following main limitations of our study have to be acknowledged. Firstly, the data is not homogeneous in regard to the allergy tests performed concerning the number of allergens tested in the individual study subjects originating from different geographic regions (Spain, Italy). This is furthermore complicated by the fact that no direct comparison is possible with previous epidemiological studies due to substantial differences in methodology among them. Secondly, many of the phenomena observed in our study cohort regarding sensitization prevalence, level and pattern are based on the molecular mechanisms of allergy. These can only be speculated upon in regard to the discussion of our results but proof is missing since molecular allergy diagnostics was not included in the analysis. Thirdly, as the most important limitation it has to be recognized, that clinical data in regard to the allergy diagnosis is missing in the included individuals. Due to this fact the conclusion of our investigation is limited to the message that allergic sensitizations is common in pediatric primary care patients and that these individuals should be subject to an allergy workup to assess the possible allergic origin of the symptoms they present with in order to reach a final diagnosis. The level of risk in regard to the development of allergic disease cannot be estimated based on our data, since no diagnosis was made as follow up.

We conclude that specific IgE sensitization in children with allergy-like symptoms is common and sensitization to multiple allergens occurred more often than mono-sensitization. Number of clinical symptoms at inclusion was higher in the atopic group compared to the non-atopic while the number of symptoms in the atopic group could not be correlated with number of positive allergens. Age seems to play a crucial role when estimating the prevalence of sensitization, since there is a significant positive correlation between patient age and the number of sensitizations.

Further studies assessing the correlation between the sensitization profiles presented above and the final doctor’s diagnosis are needed to assess the clinical relevance of the findings presented above.
